# Hepatic blood flow velocity before and 3 months after Kasai portoenterostomy is a prognostic indicator for native liver survival in biliary atresia

**DOI:** 10.1007/s00431-026-06954-y

**Published:** 2026-04-27

**Authors:** Jan Sabo, Julian Kolorz, Lukas Schipper, Thanusiah Selvamoorthy, Ines Moraleda Gudayol, Tom Hühne, Anika Hüsing, Ramsi Siaj, Elke Lainka, Kristina Kampmann, Benas Prusinskas, Doris Franke, Marie Uecker, Ulrich Baumann, Claus Petersen, Jens Dingemann, Michael Berger, Omid Madadi-Sanjani

**Affiliations:** 1 Department of Pediatric Surgery, Essen University Hospital, Essen, Germany; 2 Institute for Medical Informatics, Biometry and Epidemiology, Essen University Hospital, Essen, Germany; 3 Department of Pediatrics II, Essen University Hospital, Essen, Germany; 4 Division of Pediatric Gastroenterology, Essen University Hospital, Essen, Germany; 5 Department of Pediatric Kidney, Liver and Metabolic Diseases, Hannover Medical School, Hannover, Germany; 6 Department of Pediatric Gastroenterology, Hepatology and Liver Transplantation, Hannover Medical School, Hannover, Germany; 7 Institute of Immunology and Immunotherapy, University of Birmingham, Birmingham, UK; 8 Department of Pediatric Surgery, Hannover Medical School, Hannover, Germany

**Keywords:** Biliary atresia, Kasai portoenterostomy, Hepatic blood flow velocity, Native liver survival, Doppler ultrasound, Pediatric liver disease

## Abstract

Biliary atresia (BA) is a progressive neonatal cholangiopathy causing fibrosis, cirrhosis, and portal hypertension. Kasai portoenterostomy (KPE) restores bile drainage and prolongs native liver survival (NLS), yet BA remains the leading cause of pediatric liver transplantation. Therefore, reliable predictors of KPE failure are needed. This study evaluated hepatic blood flow velocities (HBFV) via ultrasound as potential non-invasive predictors of NLS after KPE. We retrospectively analyzed 163 BA patients treated between 2008 and 2023 at two German tertiary centers. Standardized Doppler ultrasound measured HBFV before and 3 months after KPE. Hepatic artery velocities, resistive index (RI), and portal vein flow were correlated with liver function, cholestasis, and fibrosis grade (Ishak). Furthermore, we assessed their predictive value at 3-month follow-up for NLS at 6 and 24 months. NLS in the presented cohort was 63.58% at 6 months and 40% at 24 months follow-up. No significant associations were identified between HBFV and liver fibrosis grade or cholestasis parameters (*p* > 0.05). In BA infants with 24-month NLS, the systolic hepatic artery velocity decreased significantly from diagnosis to follow-up exam. (79.46 ± 32.14 [diagnosis] vs. 56.85 ± 18.39 [3-month follow-up]; *p* = 0.001). In BA infants who did not achieve 24-month NLS (KPE failure), the arterial resistive index was significantly increased during follow-up exam (0.76 ± 0.08 [diagnosis] vs. 0.87 ± 0.14 [3-month follow-up]; *p* < 0.001).

*Conclusion*: Doppler-derived HBFV parameters may aid in early prognostication after KPE and warrant prospective validation in larger, multicenter cohorts.

**Table:** 

**What is Known:** • *Biliary atresia (BA) is the leading cause of pediatric liver transplantation. Despite Kasai portoenterostomy (KPE), many children develop fibrosis and liver failure. Reliable, non-invasive prognostic tools for KPE failure are still lacking.*
**What is New:** • *Doppler-derived hepatic blood flow velocities (HBFV) before and after KPE can predict native liver survival (NLS). Increasing hepatic artery velocity and resistive index indicate poor outcomes, while an initial decrease in portal vein flow velocity suggests improved prognosis.*

**Supplementary Information:**

The online version contains supplementary material available at 10.1007/s00431-026-06954-y.

## Introduction

Biliary atresia (BA) is a rare infantile cholestatic liver disease of unknown cause, marked by progressive fibrosis and obliteration of the bile ducts [1]. This results in biliary cirrhosis, portal hypertension, and often end-stage liver disease. Despite improvements in surgical management—particularly Kasai portoenterostomy (KPE)—many patients ultimately require liver transplantation (LT), making BA the leading global indication for pediatric LT [2, 3]. Early diagnosis and intervention are key, as timely treatment improves native liver survival (NLS). However, predicting long-term NLS after KPE remains difficult.

Prognostic factors such as age at KPE [4], fibrosis severity [5], postoperative bilirubin levels [6, 7], and biopsy findings [8] have been evaluated. However, the ongoing demand for dependable, non-invasive techniques to evaluate liver health and forecast outcomes in BA patients remains.

Ultrasound is widely available, cost-effective, and radiation-free, making it ideal for diagnosis and monitoring in BA [9, 10]. While features like the triangular cord sign and gallbladder abnormalities aid diagnosis [11, 12], the prognostic role of hepatic hemodynamics—particularly hepatic blood flow velocity (HBFV)—remains unclear. This study investigates HBFV, measured by pulsed-wave Doppler ultrasound, as a potential predictor of NLS after KPE. While prior studies have reported associations between Doppler-derived hepatic and portal flow velocity parameters and outcomes after KPE, evidence on dynamic postoperative changes, standardized multi-center acquisition, and quantitative prognostic performance remains limited. Therefore, the aim of this study was to evaluate HBFV parameters measured before and 3 months after KPE, including delta changes, as prognostic markers of NLS at 6 and 24 months.

## Materials and methods

**Fig. 1:**
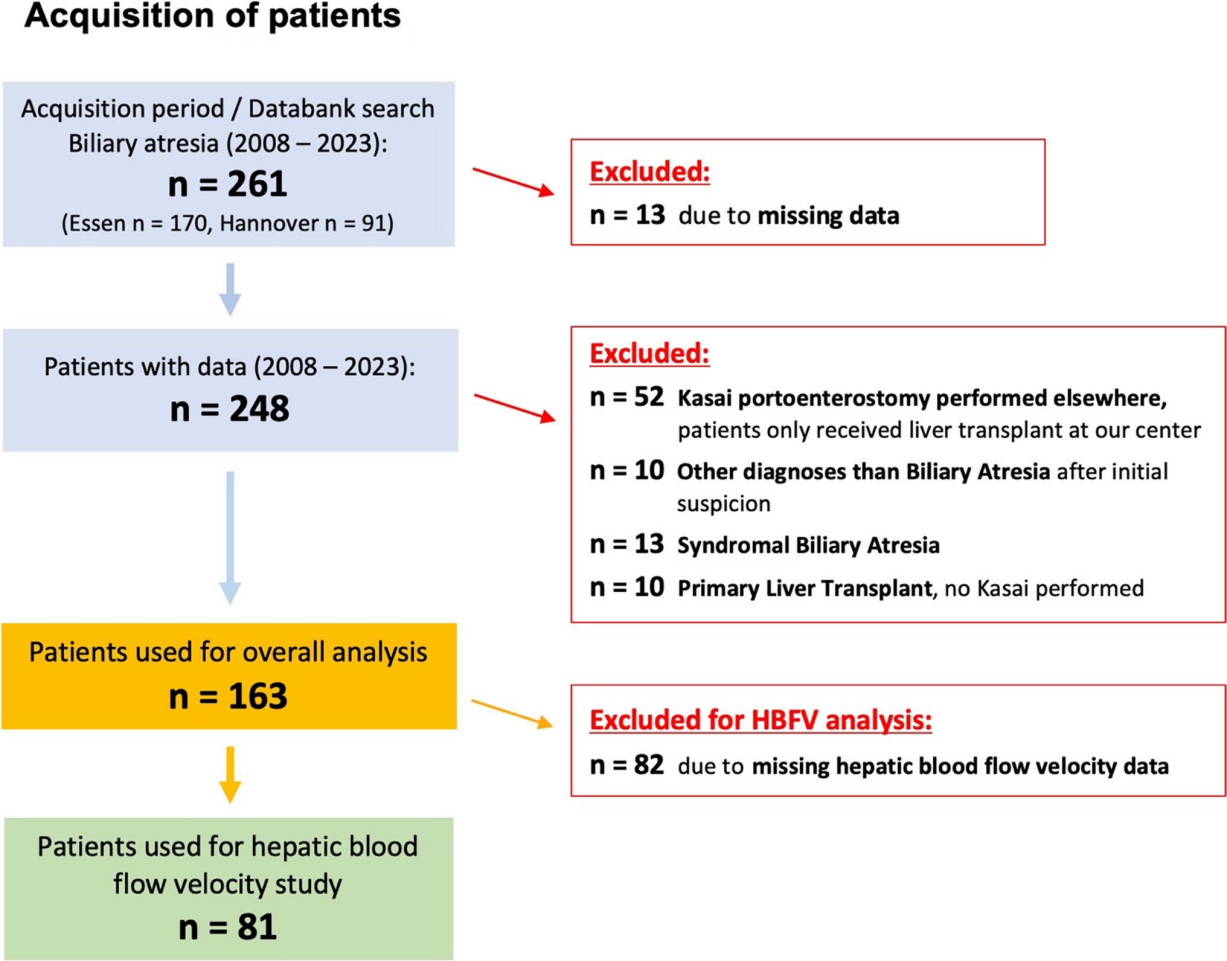
Acquisition of patients

We conducted a retrospective chart review of all biliary atresia (BA) patients diagnosed between 2008 and 2023 at two tertiary centers: Essen University Hospital (Center A) and Hannover Medical School (Center B). Inclusion criteria were isolated BA confirmed by cholangiography and/or histopathology, follow-up at 6 and 24 months post-Kasai portoenterostomy (KPE), and available Doppler ultrasound data before and 3 months after KPE. Exclusion criteria included incomplete data, syndromic BA (Biliary Atresia Splenic Malformation syndrome, BASM), primary liver transplantation, or prior KPE performed elsewhere. Among 261 identified BA patients, 163 met inclusion criteria for analysis, and 81 were eligible for hepatic blood flow velocity (HBFV) analysis (Fig. 1). Further analysis of age-at-presentation-related HBFV changes to explore the effects of fibrosis on flow velocities was limited by small subgroup sizes. NLS status at 6 months after KPE was available for all patients except one who was lost to follow-up. At 24 months, NLS status was available for 74 patients, while 7 patients were lost to follow-up.

### Fibrosis scoring

Liver fibrosis at diagnosis or KPE was graded using the Ishak scoring system (0 = no fibrosis to 6 = cirrhosis). Liver function tests (LFTs) and disease onset were evaluated across fibrosis stages to assess progression and correlation with hepatic function.

### Ultrasound investigations

Of 163 patients, 81 (49.7%) had complete data for HBFV analysis. Systolic and diastolic hepatic artery (HA) flow velocities, arterial resistive index (RI), and portal vein (PV) flow velocity were compared with LFTs, cholestasis markers, and Ishak fibrosis grade. HBFV was measured preoperatively and 3 months post-KPE. Delta changes (Δ) in HBFV parameters—ΔHAsyst (systolic HA flow velocity), ΔHAdiast (diastolic HA flow velocity), and ΔPV (portal vein flow velocity) —were analyzed for associations with native liver survival (NLS) at 6 and 24 months. Logistic regression analysis was applied to assess their predictive potential. Optimal cut-off values were determined using the Youden index. For each ROC analysis, we report the area under the curve (AUC), odds ratio (OR) with 95% confidence intervals, as well as the Youden index, sensitivity, and specificity at the selected threshold. All measurements were performed using standardized ultrasound protocols and identical equipment settings across centers to ensure data comparability. HBFV were measured using pulsed-wave Doppler ultrasound with an insonation angle maintained below 60°, and sample volumes placed centrally within the HA and PV. Peak systolic and end-diastolic velocities were recorded. The hepatic arterial RI was calculated as (peak systolic velocity—end-diastolic velocity)/peak systolic velocity. Doppler examinations were performed as part of routine clinical care and were not formally blinded to outcomes. Due to the young age of the patients, strict fasting prior to Doppler examinations was not consistently feasible. Therefore, examinations reflect routine clinical conditions.

For contextual interpretation of potential age-related hemodynamic changes, we retrospectively reviewed Doppler ultrasound examinations obtained during routine clinical care in an institutional reference cohort of infants aged 0–6 months without structural hepatobiliary disease, cholestasis, portal hypertension, or known vascular abnormalities (*n* = 22–23). Examinations were performed for transient neonatal hyperbilirubinemia (ICD P59.9) or other non-structural clinical indications and included standardized hepatic artery and portal vein Doppler measurements. These data were used exclusively for descriptive comparison with published pediatric Doppler reference values and to support interpretation of postoperative hemodynamic trends in the biliary atresia cohort. No statistical comparisons with the biliary atresia cohort or outcome analyses were performed using this reference cohort.

### Perioperative management

All KPEs were performed by dedicated pediatric hepatobiliary surgeons using laparotomy. No postoperative drainage was applied. Patients were monitored in the intensive care unit for at least 24 h, with enteral feeding initiated within 1–3 days. Postoperative care included Ursodeoxycholic acid (UDCA), fat-soluble vitamins, and a two-week intravenous antibiotic course followed by 6 months of oral prophylaxis. Since 2011, Center B introduced adjuvant steroids (budesonide 2 mg rectally daily for 3 months starting 4–7 days post-KPE), while Center A did not employ steroid therapy.

## Results

### Demographics

Data was available from 163 patients. Ninety-five patients (58.28%) were female, and 68 (41.72%) were male. In the presented cohort, NLS was 63.58% at 6 months and 40% at 24 months post-KPE. Eighty-one of these patients had complete Doppler ultrasound measurements both before and at 3 months post-KPE, and only those were included in the HBFV analysis. To assess potential selection bias, we compared baseline characteristics and outcomes between patients included in the Doppler analysis (*n* = 81) and those excluded due to missing paired Doppler data (*n* = 82). No clinically meaningful differences were observed in age at diagnosis, sex distribution, baseline laboratory parameters, or NLS at 6 and 24 months (Supplementary Figure S1).

### Time of onset compared with liver function tests (LFTs)

Diagnosis was made at a mean age of 57 days, with no gender difference. Total bilirubin averaged (11.2 mg/dl ± 4.33), conjugated bilirubin (5.99 mg/dl ± 2.52), and LFTs were increased at diagnosis (AST 176 U/I ± 176.9, ALT 118 U/I ± 108.15, gGT 442 U/I ± 356.89). Patients were grouped into four cohorts based on the days of life (d) at diagnosis: 0–29 days (group 1), 30–59 days (group 2), 60–89 days (group 2), and 90 + days (group 4). Most diagnoses occurred in groups 2 and 3. LFTs showed significant differences between groups, with later diagnosis associated with higher values. AST differed significantly between groups 2 and 4 (169 vs. 342 U/I, *p* < 0.001) and groups 1 and 3 (94 vs. 192 U/I, *p* = 0.018). ALT showed significant differences between groups 1 and 4 (49 vs. 215 U/I, *p* < 0.001) and groups 1 and 3 (49 vs. 130 U/I, *p* = 0.01). Total bilirubin showed a non-significant trend between groups 1 and 4 (9.2 vs. 14.15 mg/dl, *p* = 0.068). Conjugated bilirubin and gGT showed no significant differences between onset groups.

### Liver fibrosis grade compared with timing of BA diagnosis

No statistically significant difference was found in the distribution of fibrosis grades among the onset groups, with all grades appearing at various onset times. A slight progression of liver fibrosis with later onset diagnoses was observed in linear regression analysis.

### NLS compared with liver fibrosis grade (Ishak score) at KPE

No statistically significant difference was found in the distribution of liver fibrosis grades for either the 6-month (*p* = 0.24) or 24-month (*p* = 0.124) NLS.

### NLS compared with age at diagnosis

Despite our expectations that earlier diagnosis and treatment would lead to improved outcomes, our cohort showed no significant differences based on the timing of BA diagnosis. Importantly, patients diagnosed after 89 days showed a notable decrease in NLS, dropping from 55.56% at 6 months to 22.22% at 24 months.

## Hepatic blood flow velocity (HBFV)

### HBFVs compared with liver fibrosis grade (Ishak score)

There were no notable differences among the fibrosis grade groups (before KPE) for the HBFVs pre- and 3 months post-KPE. Nevertheless, systolic flow velocity in the hepatic artery revealed a tendency between fibrosis grades 2 and 5 (mean 54 cm/s before KPE vs. 76 cm/s before KPE; *p* = 0.077), indicating that higher fibrosis grades might be associated with increased liver stiffness and elevated peak pressures in hepatic feeding vessels. In contrast, diastolic hepatic artery flow velocity, resistive index, and portal vein flow velocity did not show any statistically significant differences.

### HBFVs compared with the time of diagnosis

No significant variations were found in the different groups of disease onset regarding hepatic artery and portal vein flow velocities. However, a statistically significant difference was observed in resistive index between group 1 (0–29 days of life) with a mean of 0.7 and groups 3 (60–89 days of life) and 4 (> 90 days of life) with means of 0.77 and 0.8, respectively, *p* = 0.046 and *p* = 0.021.

### HBFV before and 3 months after KPE compared to 6-month NLS

**Fig. 2:**
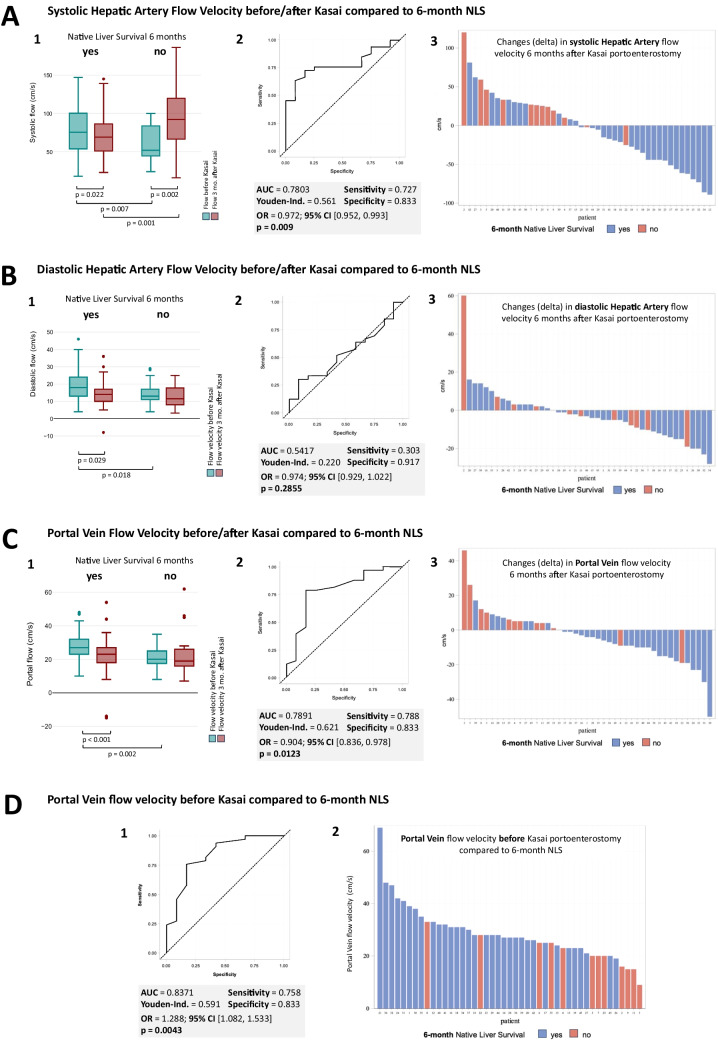
Hepatic blood flow velocities (HBFV) before and after Kasai portoenterostomy (KPE) were compared with 6-month native liver survival (NLS). **A1**, **B1**, and **C1** show box plot diagrams of HBFV before (green) and 3 months after (red) KPE conducted in *n* = 80 patients (*n* = 55 with positive NLS and *n* = 25 with negative NLS), each compared with 6-month NLS. Boxplots show median (line), interquartile range (box), whiskers represent the range, and dots mark outliers. Significant changes between boxplots are shown by *p*-values. **A2**, **B2**, and **C2** show logistic regression results conducted in *n* = 45 patients, with ROC curves for each flow velocity delta variable, along with their AUC, Youden index, sensitivity, specificity, odds ratio with 95% confidence interval, and significance (*p*-value) for predicting NLS at 6 months post-KPE. **A3**, **B3**, and **C3** show bar charts from *n* = 45 patients with HBFV changes (deltas) 3 months after KPE for each patient. The logistic regression analysis results indicate significant associations with changes in systolic HA flow velocity (AUC 0.7803, *p* = 0.009) and Portal Vein flow velocity (AUC 0.7891; *p* = 0.0123), both of which demonstrate reduced flow velocity after KPE among patients with a positive 6-month NLS outcome. **D** displays the Portal Vein flow velocity prior to KPE (at diagnosis) compared to 6-month NLS in *n* = 47 patients. The ROC curve in (**D1**) illustrates the predictive accuracy of Portal Vein flow velocity at diagnosis for 6-month NLS. **D2** presents individual portal vein flow values at diagnosis, along with their 6-month NLS status, with blue indicating NLS and red indicating no NLS. Generally, higher initial Portal Vein flow velocities before KPE correlate with a greater likelihood of 6-month NLS survival (AUC = 0.8371; *p* = 0.0043). AUC, area under the curve; HA, hepatic artery; HPFV, hepatic blood flow velocity; KPE, Kasai portoenterostomy; NLS, native liver survival; RI, resistive index; OR, odds ratio; CI, confidence interval

Patients with a 6-month NLS showed decreased systolic (81 cm/s before KPE to 67 cm/s 3 months after KPE, *p* = 0.022) and diastolic (18 cm/s before KPE to 13 cm/s 3 months after KPE, *p* = 0.029) flow velocities (Table 1 and Fig. 2). In contrast, children with KPE failure within 6 months showed increased systolic flow velocity (52 cm/s before KPE to 101 cm/s 3 months after KPE, *p* = 0.002) but unchanged diastolic flow velocity. The resistive index rose only in the group with KPE failure (0.76 before KPE to 0.87 3 months after KPE, *p* < 0.001). Portal vein flow velocity decreased in the NLS group (27 cm/s before KPE to 23 cm/s 3 months after KPE, *p* < 0.001), but remained unchanged in the KPE failure group.

**Table 1:** HBFVs and Resistive Index (RI) before and after Kasai portoenterostomy (KPE) compared with 6-month and 24-month Native Liver Survival (NLS). Data are presented as median (mean ± SD [range]) and significance is shown by *p*-values. In the 6-month NLS evaluation of HBFV, 80 patients qualified, comprising 55 with a positive NLS and 25 with a negative NLS. For the 24-month NLS assessment, 74 patients were eligible, including 30 with a positive NLS and 44 with a negative NLS

**Native liver survival**	**NLS 6 months yes**		
Number of patients	55 (68.75%)		*p*-value
	**Before KPE**	**3 months after KPE**	
Systolic hepatic artery flow velocity (cm/s)	**81** (80.31 ± 33.27 [18–147])	**67** (67.06 ± 25.26 [26–145])	**0.022**
Diastolic hepatic artery flow velocity (cm/s)	**18** (19.11 ± 8.85 [4–40])	**13** (14.07 ± 7.32 [−8–36])	**0.029**
Resistive index	**0.76** (0.76 ± 0.07 [0.63–0.89])	**0.77** (0.78 ± 0.11 [0.42–1.1])	ns
Portal vein flow velocity (cm/s)	**27** (28.82 ± 10.05 [10–69])	**23** (20.94 ± 13.06 [−23–54])	**<0.001**
**Native liver survival**	**NLS 6 months no**		
Number of patients	25 (31.25%)		*p*-value
	**Before KPE**	**3 months after KPE**	
Systolic hepatic artery flow velocity (cm/s)	**52** (58.38 ± 22.98 [24–100])	**101** (95.33 ± 43.64 [16–186])	**0.002**
Diastolic hepatic artery flow velocity (cm/s)	**12** (13.87 ± 6.4 [4–29])	**10** (14.14 ± 14.26 [3.2–71])	ns
Resistive index	**0.76** (0.76 ± 0.09 [0.57–0.9])	**0.87** (0.9 ± 0.14 [0.75–1.4])	**<0.001**
Portal vein flow velocity (cm/s)	**20** (21.33 ± 7.16 [8–35])	**19** (22.86 ± 13.41 [7–62])	ns
**Native liver survival**	**NLS 24 months yes**		
Number of patients	30 (40.54%)		*p*-value
	**Before KPE**	**3 months after KPE**	
Systolic hepatic artery flow velocity (cm/s)	**81** (79.46 ± 32.14 [18–147])	**52** (56.85 ± 18.39 [26–95])	**<0.001**
Diastolic hepatic artery flow velocity (cm/s)	**19** (19.72 ± 9.56 [4–40])	**13** (14.13 ± 6.7 [5.4–36])	**0.013**
Resistive index	**0.76** (0.76 ± 0.07 [0.63–0.88])	**0.74** (0.74 ± 0.1 [0.42–0.9])	ns
Portal vein flow velocity (cm/s)	**27** (28.81 ± 8.44 [10–48])	**24** (23.5 ± 7.26 [11–44])	**0.017**
**Native liver survival**	**NLS 24 months no**		
Number of patients	44 (59.46%)		*p*-value
	**Before KPE**	**3 months after KPE**	
Systolic hepatic artery flow velocity (cm/s)	**56** (64.15 ± 26.67 [24–130])	**83** (88.45 ± 38.64 [16–186])	**0.004**
Diastolic hepatic artery flow velocity (cm/s)	**14** (15.12 ± 6.67 [4–33])	**11** (13.84 ± 11.93 [−8–71])	ns
Resistive index	**0.76** (0.76 ± 0.08 [0.57–0.9])	**0.84** (0.87 ± 0.14 [0.7–1.4])	**<0.001**
Portal vein flow velocity (cm/s)	**24** (32.19 ± 7.49 [8–39])	**19** (19.84 ± 14.64 [−23–62])	ns

### Portal vein flow velocity before KPE compared with 6-month NLS

The logistic regression analysis of each patient's portal vein flow velocity before KPE reveals a highly significant difference (*p* < 0.0043) and a predictive value for NLS at 6 months post-KPE of AUC = 0.8371 (Fig. 2 panel D1). The higher the initial flow velocities before KPE, the higher the probability of a positive 6-month NLS outcome (Fig. 2 panel D2).

### HBFVs before and 3 months after KPE compared with 24-month NLS

**Fig. 3:**
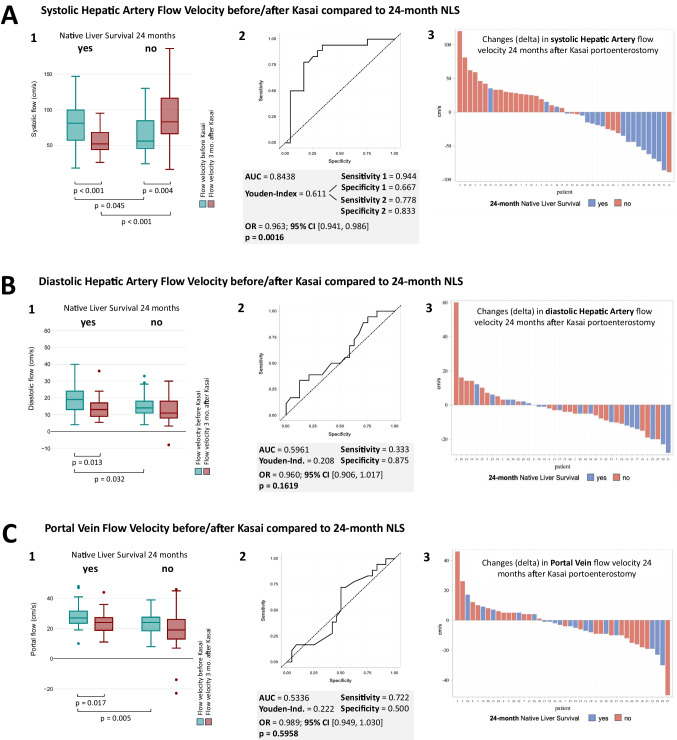
Hepatic blood flow velocities (HBFV) in 74 patients before and after Kasai portoenterostomy (KPE) were compared with 24-month native liver survival (NLS). **A1**, **B1**, and **C1** show box plot diagrams of HBFV before (green) and 3 months after (red) KPE conducted in *n* = 74 patients (*n* = 30 with positive NLS and *n* = 44 with negative NLS), each compared with 24-month NLS. Boxplots show median (line), interquartile range (box), whiskers represent the range, and dots mark outliers. Significant changes are shown by p-values. **A2**, **B2**, and **C2** show logistic regression results conducted in *n* = 42 patients with ROC curves for each flow velocity delta variable, along with their AUC, Youden index, sensitivity, specificity, odds ratio with 95% confidence interval, and significance (*p*-value) for predicting NLS at 24 months post-KPE. **A2** specifically shows two optimal cut-offs with identical Youden indices but different optimal sensitivities and specificities. **A3**, **B3**, and **C3** show bar charts from *n* = 42 patients with HBFV changes (deltas) 3 months after KPE for each patient. The logistic regression analysis results indicate a significant association with changes in systolic HA flow velocity only (AUC 0.8438, *p* = 0.0016), showing reduced flow velocity after KPE among patients with a positive 24-month NLS and increased flow velocity among those with a negative 24-month NLS outcome. AUC, area under the curve; HA, hepatic artery; HPFV, hepatic blood flow velocity; KPE, Kasai portoenterostomy; NLS, Native Liver Survival; RI, resistive index; OR, odds ratio; CI, confidence interval

The results at 24 months were consistent with those at 6 months (Table 1 and Fig. 3). In patients with NLS, there was a significant decrease in systolic (from 81 cm/s before KPE to 52 cm/s 3 months after KPE, *p* = 0.001) and diastolic flow velocity (from 19 cm/s before KPE to 13 cm/s 3 months after KPE, *p* = 0.013). Conversely, children with KPE failure exhibited an increase in systolic flow velocity (from 56 cm/s before KPE to 83 cm/s 3 months after KPE, *p* = 0.004), with diastolic flow velocity remaining stable. The resistive index rose in the KPE failure group (from 0.76 before KPE to 0.84 3 months after KPE, *p* < 0.001), while it stayed the same in the NLS group. Portal vein flow velocity decreased in both groups (27 cm/s before KPE to 24 cm/s 3 months after KPE, *p* < 0.017, and 24 cm/s before KPE to 20 cm/s 3 months after KPE without significance, respectively).

### HBFV ‘deltas’ compared with 6-month NLS

HBFV delta calculations for 6-month NLS were conducted in 45 patients. ROC-derived thresholds, AUC, sensitivity, specificity, OR, and its 95% CI are provided for each HBFV delta parameter in Fig. 2. Logistic regression analysis of the systolic hepatic artery delta showed a significant difference (*p* = 0.009) with a predictive value of AUC = 0.7803 (Fig. 2, panel A2). A decrease in systolic hepatic artery flow velocity was predictive of 6-month NLS, with only one exception (Fig. 2 panel A3). The diastolic hepatic artery flow velocity delta was not significant (Fig. 2, panel B2). The portal vein flow velocity delta also showed significant differences (*p* = 0.0123) with a predictive value of AUC = 0.7891 (Fig. 2, panel C2). A decrease in portal vein flow velocity was associated with 6-month survival, except for two patients (Fig. 2, panel C3). Increasing flow velocity values were observed in both NLS and non-NLS patients for both the systolic hepatic artery and portal vein deltas (Fig. 2, panels A3 and C3).

### HBFV ‘deltas’ compared with 24-month NLS

HBFV delta calculations for 24-month NLS were performed in 42 patients. ROC-derived thresholds, AUC, sensitivity, specificity, OR, and its 95% CI are provided for each HBFV delta parameter in Fig. 3. Logistic regression analysis of the systolic hepatic artery delta revealed a significant difference (*p* = 0.0016) with a predictive value of AUC = 0.8438 (Fig. 3, panel A2). As with the 6-month NLS analysis, a decrease in systolic hepatic artery flow velocity was predictive of 24-month NLS, whereas an increase was predictive of KPE at 24 months (Fig. 3, panel A3). Delta analyses of the diastolic hepatic artery and portal vein proved insignificant, with negligible predictive values (AUC = 0.5961 and 0.5336, respectively) (Fig. 3, panels B2 and C2).

### Institutional reference cohort

To assess potential physiological age-related hemodynamic changes during early infancy, Doppler measurements from an institutional reference cohort of infants aged 0–6 months without structural hepatobiliary disease were analyzed (n = 22–23) (Supplementary Figure S2). No significant association between age and systolic HA peak velocity was observed (*p* = 0.93). Similarly, no significant age-dependent associations were found for diastolic HA velocity (*p* = 0.60), hepatic arterial resistive index (*p* = 0.75), or portal vein flow velocity (*p* = 0.27). These findings suggest that Doppler-derived hepatic and portal flow velocities remain relatively stable during the first 6 months of life.

## Discussion

Despite advances in the management of pediatric hepatopathies, the mortality rate for children on the liver transplant waiting list remains high [13]. This is particularly concerning in the context of biliary atresia (BA), where early surgical intervention with Kasai portoenterostomy (KPE) often delays, but does not eliminate the need for liver transplantation (LT). Timely identification of children with KPE failure requiring early listing for transplantation is therefore of paramount clinical importance. The current standard of care lacks robust non-invasive tools capable of reliably predicting clinical deterioration in this vulnerable population. As a result, clinical decisions such as transplant listing or preparation for living donor liver transplantation (LDLT) are often reactive rather than proactive.

Non-invasive imaging modalities are central in monitoring children with chronic liver disease. Among these, ultrasound offers a cost-effective, accessible, and radiation-free approach to serial monitoring. When performed by experienced investigators, it provides high reproducibility, particularly for Doppler-based assessments [14]. However, routine ultrasound examinations have traditionally emphasized indirect or secondary signs of progressive disease—such as splenomegaly, collateral vessel formation, portal hypertension, and ascites—as well as static morphologic liver changes. Additionally, newer elastography techniques (e.g., transient elastography or 2D-shear-wave elastography) have been introduced to assess liver stiffness non-invasively [15]. However, their interpretation in pediatric patients is still fraught with challenges [16]. Although elastography is increasingly being adopted as a standard modality in children, measurements may still be influenced by inflammation, cholestasis, and technical factors, which should be taken into account when interpreting prognostic estimates.

Our study aimed to specifically examine the role of Doppler-based hepatic blood flow velocities (HBFV) in predicting clinical outcomes in children with BA after KPE. Particularly, we investigated whether changes in the hepatic artery (HA) and portal vein (PV) velocities could serve as surrogate markers for native liver survival (NLS), based on the hypothesis that livers with more advanced fibrosis, which causes increased stiffness, would show higher blood flow velocities due to increased resistance, while those with better elasticity (i.e., less fibrosis) would display lower velocities.

Studies in adults with chronic liver disease have documented increased hepatic resistance and altered portal and arterial flow patterns in the context of fibrosis and cirrhosis [17–19]. However, pediatric studies are lacking, particularly in terms of outcome correlation in BA patients. El Guindi et al. described Doppler flow parameters in BA patients primarily for diagnostic rather than prognostic purposes [9]. Wanek et al. reported on portal venous velocity in a small BA cohort, concluding that decreasing PV velocity may be an indicator for the necessity of LT [20]. In accordance with these results, Khong et al. reported on the association of decreasing portal venous velocities and the timing of transplant [21]. Kardorff et al. demonstrated that children with post-KPE diminished maximum portal flow velocity, flattened hepatic vein flow curve, and a high hepatic artery resistance index showed lower levels of hepatic protein synthesis and were therefore associated with a higher disease severity [22]. In contrast to previous studies that primarily reported static Doppler associations at single time points, the present study focuses on dynamic postoperative changes in HBFV and their prognostic relevance at defined early follow-up intervals. This trajectory-based approach provides additional information beyond absolute values and cohort size alone. Classic surgical outcome studies have identified early postoperative clinical and biochemical markers as key predictors of NLS after KPE. In particular, Superina et al. demonstrated that early postoperative bilirubin clearance strongly correlates with long-term NLS and remains a cornerstone for postoperative risk stratification [23]. In this context, our Doppler-derived hemodynamic findings should be interpreted as complementary rather than competitive, providing additional insight into intrahepatic vascular adaptation beyond established biochemical predictors.

Despite the results from small BA patient groups, our study is among the largest BA cohorts examining the predictive value of hepatic blood flow velocities for KPE outcome, confirming the predictive value for later KPE outcome. Within this framework, our Doppler ultrasound findings offer a practicable tool for predicting outcomes. One of the study’s most striking results is the significant and opposite trend in systolic HA velocities in patients with NLS compared to non-NLS at both 6 and 24 months after KPE. Patients with NLS demonstrated a decrease in systolic HA flow velocity compared to preoperative levels, while patients with non-NLS showed significant increases. This yielded a high predictive value for 24-month survival (AUC = 0.8438). The delta change of systolic HA velocity was particularly consistent, with increasing velocities strongly associated with non-NLS. This pattern supports the hypothesis that increasing fibrotic resistance raises HA velocity. Interpretation of postoperative changes in HA velocity requires consideration of physiological cardiovascular maturation during infancy. Published pediatric Doppler reference data, including Verhagen et al. [24], demonstrate relatively stable HA peak systolic velocities throughout early infancy, without evidence of a pronounced age-dependent decline. Consistently, our institutional reference cohort (0–6 months) showed only a minimal, statistically non-significant age-related trend, indicating that physiological maturation explains little variability in HA velocity during this interval. If the observed postoperative reduction were primarily attributable to maturation, a similar directional pattern would be expected in both BA outcome groups. Instead, we observed divergent trajectories, with a decrease in the NLS group and a marked increase in the poor outcome group. This opposing directionality supports the interpretation that postoperative HA velocity changes reflect outcome-specific vascular remodeling rather than normal developmental adaptation alone. These findings echo the work of El-Guindi et al., who reported dilated hepatic arteries in BA patients at diagnosis, likely due to hypertrophy driven by intrahepatic resistance [9]. Jeon et al. further demonstrated that hepatic artery diameter decreased in native liver survivors but increased in patients requiring transplant, corroborating our conclusions [10]. Importantly, our data indicate that preoperative Doppler-derived parameters alone are insufficient to reliably predict 24-month NLS. The primary prognostic value of Doppler ultrasound in this context lies in early postoperative hemodynamic trajectories rather than isolated baseline measurements. Postoperative bilirubin clearance remains one of the strongest predictors of outcome after KPE. However, Doppler-derived HBFV reflect intrahepatic vascular resistance and hemodynamic adaptation rather than bile drainage alone, and may therefore provide complementary information, particularly in patients with equivocal biochemical responses.

Similarly, our data showed a rise in the HA resistance index among non-survivors, while it remained stable in survivors. This finding reinforces the view that increased resistance in fibrotic livers affects arterial hemodynamics and could serve as a prognostic indicator. While we observed a statistically significant decrease in postoperative portal vein flow velocities in patients with NLS, both at 6 and 24 months, these results diverge from adult studies, where reduced PV flow velocity is often associated with worse outcomes due to cirrhosis-related resistance [23]. Our pediatric cohort showed better results with lower PV flow velocity, especially when the flow decreased from higher rates, likely due to the reversible nature of inflammation after KPE and the state of the portal vein (normoplastic vs. hypoplastic). Pediatric livers, especially in neonates, may experience initially elevated PV flow velocity due to acute inflammation. Successful KPE could normalize this hyperdynamic state. Thus, a decrease in PV flow may signify a return to more physiologic perfusion. However, this interpretation should be approached cautiously, as postoperative PV flow changes may reflect normalization from a hyperdynamic inflammatory state rather than a direct surrogate of fibrosis severity or portal hypertension. The physiological reason for this finding likely lies in developmental differences in neonatal and adult livers, as the neonates have a higher regenerative capacity and adaptability to inflammation and fibrosis.

Retrospective analysis inherently introduces selection bias and lacks control over key confounding variables. Although no clinically relevant differences were observed between included and excluded patients, the requirement for paired preoperative and 3-month Doppler examinations may still introduce selection bias, as patients with more structured follow-up or technically feasible Doppler measurements may be overrepresented. Furthermore, Doppler assessments were intentionally limited to diagnosis and 3 months after KPE to capture early postoperative hemodynamic adaptation while minimizing confounding from progressive fibrosis or recurrent cholangitis. Intermediate or later measurements (e.g., at 6 or 12 months) were not consistently available in this retrospective cohort and could therefore not be analyzed, representing a limitation of the study. Postoperative management differed between centers, particularly with regard to the use of adjuvant corticosteroid therapy. Corticosteroids may influence postoperative inflammation, edema, and hepatic perfusion, and thus could affect Doppler-derived hemodynamic parameters independent of surgical success. Due to the retrospective design and limited center-specific sample sizes, we were unable to formally adjust for this potential confounder. Additionally, the small subgroup sizes prevented analysis of age-at-presentation-dependent differences in HBFV changes and examination of the effect of various liver fibrosis stages. This underscores the necessity for large, multicenter studies to explore this possible influence on flow velocities. Sobriety may lead to changes in HBFV due to changes in the splanchnic blood circulation, and it was not always strictly maintained during ultrasound examinations. However, recent studies indicate that being sober isn't necessarily a major factor for bias, at least in diagnosing biliary atresia [25]. Another limitation of this study is the restricted follow-up duration. Although NLS at 24 months represents a clinically meaningful endpoint, longer-term outcomes beyond this period could not be robustly assessed due to incomplete longitudinal Doppler follow-up. Early postoperative hemodynamic changes may reflect ongoing vascular remodeling and fibrotic progression. However, their long-term prognostic implications require prospective validation as no independent validation cohort was available. Missing or inconsistent data are also risks in such study designs. Nonetheless, we attempted to mitigate these effects through rigorous statistical methods, data validation, and cross-referencing. Although patients from two tertiary German centers were recruited for the analysis, a multi-center, prospective study is necessary to validate our findings across diverse patient populations and clinical settings. Only through collaborative efforts can we identify reproducible prognostic parameters for BA outcomes.

In conclusion, this study provides important insights into the prognostic relevance of HBFVs in infants with biliary atresia undergoing KPE. Our analysis demonstrated three key findings: (1) a significant inverse correlation between systolic hepatic artery flow velocity and NLS at both 6 and 24 months, (2) a postoperative decrease in portal vein flow velocities with NLS, and (3) a notable predictive value of preoperative portal vein flow velocity for early NLS outcomes. These findings emphasize the unique hemodynamic patterns in pediatric biliary atresia, diverging from the well-established pathophysiology of adult liver diseases. Although the results must be interpreted with caution owing to the retrospective design and cohort size, and considering that the standard reference ranges for pediatric HBFV and resistive indices vary with age and have not been systematically established, they highlight the potential of Doppler ultrasound as a non-invasive and accessible tool in early prognostication management. Further validation in prospective, multi-center studies is warranted to confirm these associations and integrate blood flow metrics into routine clinical decision-making, with the goal of improving outcomes and guiding timely interventions for children affected by this complex disease.

## Supplementary Information

Below is the link to the electronic supplementary material.

ESM 1 (PDF 391 KB)

## Data Availability

No datasets were generated or analysed during the current study.

## Abbreviations

ALT: Alanin-transferase
AST: Aspartat-transferase
AUC: Area under the curve
BA: Biliary atresia
BASM: Biliary atresia splenic malformation
CI: Confidence interval
gGT: Gamma-glutamyl-transferase
HA: Hepatic artery
HBFV: Hepatic blood flow velocity
KPE: Kasai portoenterostomy
LDLT: Living donor liver transplantation
LFT: Liver function test
LT: Liver transplantation
NLS: Native liver survival
OR: Odds ratio
PV: Portal vein
RI: Resistive index
UDCA: Ursodeoxycholic acid
