# Supplementary material for: Hepatic blood flow velocity before and 3 months after Kasai portoenterostomy is a prognostic indicator for native liver survival in biliary atresia
Source: Eur J Pediatr. 2026 Apr 27;185(5):304. doi: 10.1007/s00431-026-06954-y (PMC13121209; doi:10.1007/s00431-026-06954-y)
Supplement: Supplementary file 1 — ESM 1 (PDF 391 KB) [file 431_2026_6954_MOESM1_ESM.pdf]

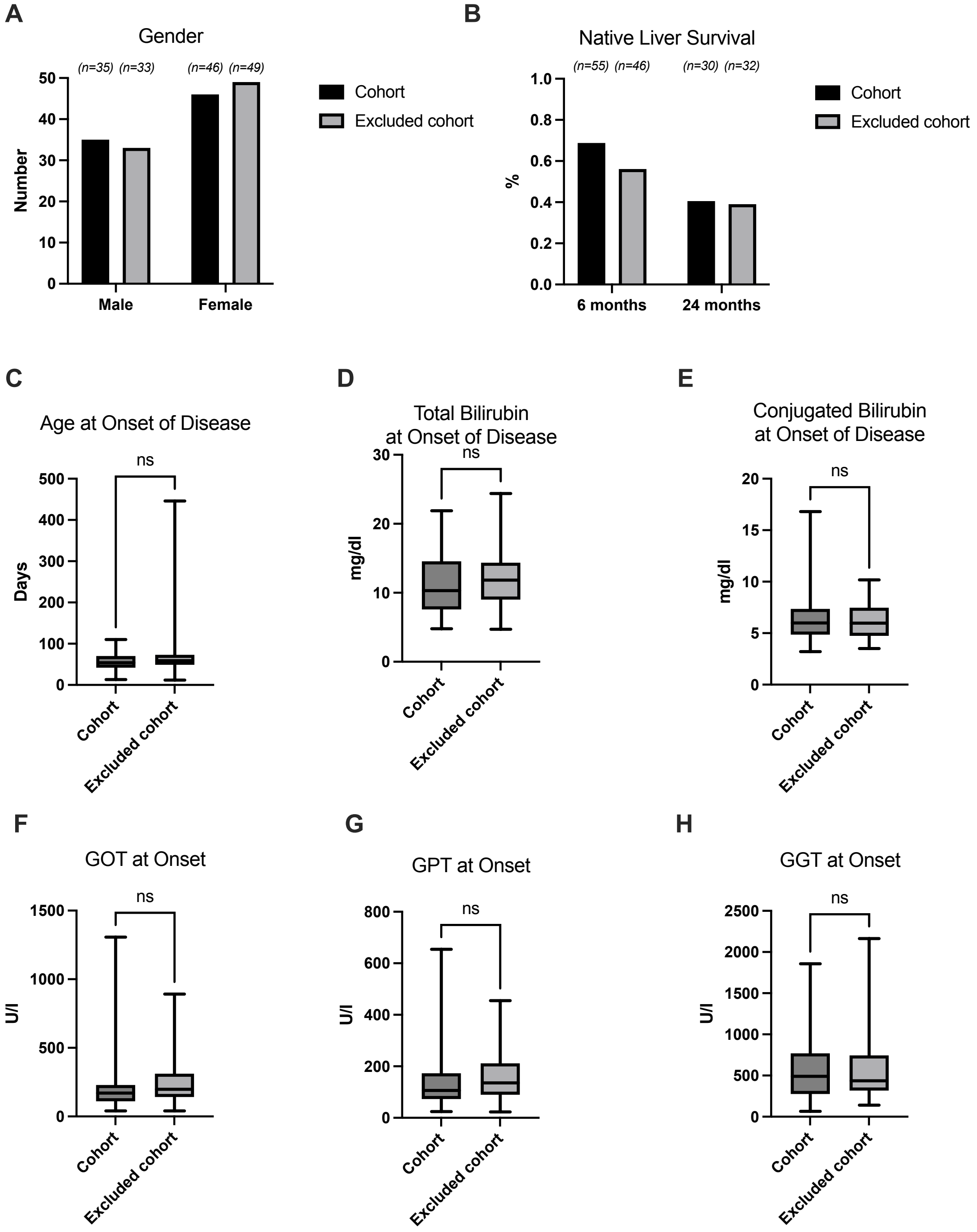

Supplementary Figure S1. **Comparison of baseline characteristics and outcomes between included and excluded patients (A-H).** Baseline demographic characteristics, laboratory parameters at disease onset, and outcomes were compared between the included cohort (n = 81) and excluded patients (n = 82). Shown are sex distribution (A), age at onset of disease (C), total and conjugated bilirubin (D-E), GOT, GPT, and GGT levels (F-H) at disease onset, as well as native liver survival at 6 and 24 months (B). Continuous variables are displayed as box-and-whisker plots, with the box indicating the median and interquartile range and whiskers representing minimum and maximum values. Categorical variables are presented as absolute numbers or proportions. Group comparisons were performed using the Mann–Whitney U test for continuous variables and χ^2 or Fisher's exact test for categorical variables, as appropriate. No statistically significant differences were observed between groups (ns).

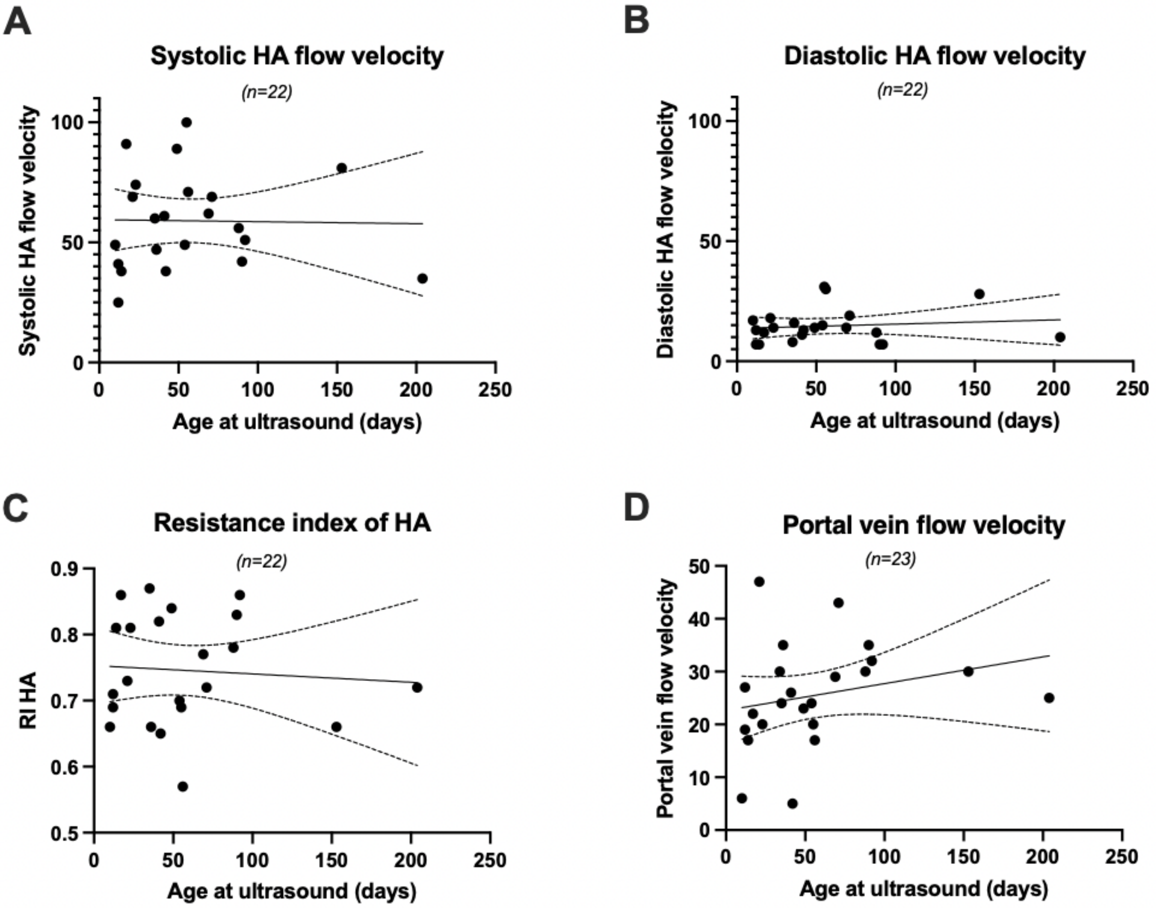

Supplementary Figure S2. **Age-dependent analysis of Doppler-derived hepatic and portal flow velocities in infants without structural hepatobiliary disease (0–6 months)**. Scatter plots with simple linear regression of systolic hepatic artery (HA) velocity (A), diastolic HA velocity (B), hepatic arterial resistive index (RI) (C), and portal vein velocity (D) (n = 22–23). No significant age-dependent association was observed for systolic HA velocity, diastolic HA velocity, RI, or portal vein velocity (all $p > 0.25$), indicating that Doppler-derived hepatic and portal flow parameters remained relatively stable during early infancy in this reference cohort.
